# Translation, Cultural Adaptation, and Psychometric Properties of the Life Satisfaction Index for the Third Age—Short Form (LSITA-SF12) for Use among Ethiopian Elders

**DOI:** 10.3390/nursrep11040089

**Published:** 2021-12-07

**Authors:** Habtamu Sewunet Mekonnen, Helena Lindgren, Biftu Geda, Telake Azale, Kerstin Erlandsson

**Affiliations:** 1 Department of Medical Nursing, School of Nursing, College of Medicine and Health Sciences, University of Gondar, Gondar 196, Ethiopia; 2 Department of Women’s and Children’s Health, Karolinska Institutet, 171 77 Solna, Sweden; helena.lindgren@ki.se (H.L.); ker@du.se (K.E.); 3 Department of Nursing, College of Health Science, Shashamene Campus, Madda Walabu University, Robe 247, Ethiopia; biftug@gmail.com; 4 Department of Health Education and Behavioral Sciences, Institute of Public Health, College of Medicine and Health Sciences, University of Gondar, Gondar 196, Ethiopia; atelake07@gmail.com; 5 School of Education, Health and Social Studies, Dalarna University, 791 31 Falun, Sweden

**Keywords:** cultural adaptation, elderly, life satisfaction, northwest Ethiopia, psychometric test

## Abstract

(1) Background: Self-reported measures play a crucial role in research, clinical practice, and health assessment. Instruments used to assess life satisfaction need validation to ensure that they measure what they are intended to detect true variations over time. An adapted instrument measuring life satisfaction for use among Ethiopian elders was lacking; therefore, this study aimed to culturally adapt and evaluate the psychometric properties of the Life Satisfaction Index for the Third Age—Short Form (LSITA-SF12) in Ethiopia. (2) Methods: Elderly people (*n* = 130) in Metropolitan cities of northwestern Ethiopia answered the LSITA-SF12 in the Amharic language. Selected reliability and validity tests were examined. (3) Result: The scale had an acceptable limit of content validity index, internal consistency, test-retest, inter-rater reliabilities, and concurrent and discriminant validities. (4) Conclusion: The Amharic language version of LSITA-SF12 appeared to be valid and reliable measures and can be recommended for use in research and clinical purposes among Amharic-speaking Ethiopian elders.

## 1. Introduction

Nowadays, people live longer because of urbanization, industrial development, advances in science, technology, and the modern way of life [1]. Attributed to these rapid demographic changes, by 2050, the world’s and Africa’s older population is expected to be 2 billion and 225.8 million, respectively [2,3,4]. In Ethiopia, migration to urban areas for work, family support, and medical care increasingly brings elders to the cities [5].

Life satisfaction is a vital indicator of living standards, and it is critical to the social and economic engagement of older people. Life Satisfaction is defined as a feeling of satisfaction with how one has lived his/her life up to that point and a state of being content with one’s current life. Successful aging and life satisfaction are synonymous terms [6]. In addition, satisfaction with life is a great success story of elders, and it is the reflection of human development, longer lives, healthy aging, and full adaptation/success to aging [7]. When the elders are satisfied with their life, they will lead their aging successfully [8].

Improving measurement, monitoring, and research on Healthy Ageing is one of the five world health organization’s (WHO) strategic objectives of global strategy and action plan on aging and health [9]. In addition, one of the directions of the Madrid International Plan of Action on Ageing and the WHO active aging framework is to make cities age-friendly, which is a necessary and logical response to promote the well-being and contributions of older urban residents and keep cities prosperous [10].

Similarly, the Ethiopian national plan of action on older persons recommends the research of the social, economic, and psychological conditions of older persons in the country. In addition, it suggests the cooperation of educational and research institutions of the federal and regional governments on the specific issues of older persons [1]. However, finding an appropriate tool to research the intended population might be difficult. Additionally, the measurement tools may not be adequately measured and bring the desired outcomes. Hence, looking over the various sources is necessary.

Generally, choosing the most valid and reliable tool and making it fit with the proposed concept are considered significant components of this research work. The authors have reviewed the literature and selected Life Satisfaction Index for the Third Age—Short Form (LSITA-SF12) for the current study because, the LSITA-SF12 scale is developed and validated in the English language among elderly people, but most of the other scales were developed for a general population. In addition, this scale measures the overall construct of life satisfaction which is not domain specific and has an excellent reliability with satisfactory content, construct, and criterion validity. Globally, there are life satisfaction measurement scales adapted to different cultures. However, as to the authors’ best knowledge, there is no available validated and culturally adapted measurement tool of life satisfaction for the Ethiopian elders.

Developing the Amharic version of the scale will help as a tool for future researches in Ethiopia on life satisfaction. The finding will be a baseline and an input for the national plan of action for elderly populations. Therefore, the study aimed to culturally adapt and investigate the psychometric properties of the Life Satisfaction Index for the Third Age—Short Form (LSITA-SF12) for use among Ethiopian elders.

### 1.1. Statement of Significance

#### 1.1.1. Problem or Issue

Despite the significance of validated measurement tools for measurement or research work, an adapted and validated instrument measuring life satisfaction among Ethiopian elders was lacking.

#### 1.1.2. What Is Already Known

There are life satisfaction assessment scales validated in various countries and languages. The Life Satisfaction Index for the Third Age—Short Form (LSITA-SF12) is one of the scales developed and validated in the English language. This scale is intended to measure the life satisfaction of elderly people.

#### 1.1.3. What This Paper Adds

The Life Satisfaction Index for the Third Age—Short Form (LSITA-SF12) was translated in to Amharic language and culturally adapted to Ethiopian Elderly people. The scale found adequate psychometric properties and it can be the choice for use among Amharic-speaking Ethiopian elders.

## 2. Methods and Materials

### 2.1. Study Design, Period, and Setting

The study design was a community-based cross-sectional study. The study was conducted from 22 October 2020 to 30 November 2020 in metropolitan cities of northwest Ethiopia. In northwest Ethiopia, there are two metropolitan cities, Gondar and Bahir Dar administrative cities. Bahir Dar has an estimated older people of 13,792 aged 60 years and above. Gondar is a royal and ancient historical city of Ethiopia. It is found in Central Gondar zone, Amhara regional state. It is located at 175.5 km from Bahir Dar and has an estimated 9556 people aged 60 and above.

### 2.2. Sample and Data Collection

Elderly people aged ≥60 years and have lived for six months and above in Metropolitan cities of northwestern Ethiopia during the study period were the source population. Those elders who were present during the specified data collection period were the study population. Elderly people whose ages were ≥60 years and who were residents of metropolitan cities of northwestern Ethiopia were included in the study, yet individuals who were living in streets, religious institutions, and temporary settlements were excluded. The sample size was determined by considering eleven subjects per item ratio (130 elderly people) [11,12]. The elderly participants were selected using purposive sampling. Data collectors were trained and well-informed about the data collection process and the variations in age, literacy, health condition, socioeconomic status, and other characteristics of the study population. To this end, both male and female participants ranged from young–old to old–old, unable to read and write to first degree, very good to very bad perceived health status, and low to high income. The scale is intended to be used for all Amharic speaking Ethiopian elders. In addition, these variations are very important factors which affect the life satisfaction of the elderly. Thus, considering such variations during the scale validation is of paramount importance and strengthens the usefulness of the scale.

Before the data collection, there was communication with the elderly associations, focal person/s of elderly affairs, and health extension workers in each city. Then, with the help of these people (workforces), the participants were reached. An interviewer-administered structured questionnaire was used for gathering data. Seventy elders from Bahr Dar city and sixty from Gondar city were selected considering the number of estimated elderly population in each city. Data were collected by data collectors; six B.Sc. nurses were supervised by two M.Sc. nurses. The overall events were managed and supervised by the principal investigator. To ensure the quality of data, a two-day training was provided in each city for data collectors and supervisors about the data collection tool and data collection procedures. Participants were interviewed in quiet areas of their homes after the data collectors briefly explained the study purpose and got consent from each participant.

Written informed consent was obtained from study participants before the interview. For participants who could not read and write, a thumbprint was used in place of the participants’ signature. Each study participant was informed about the purpose, method, expected benefit, and risk of the study. They were also informed about their full right not to participate or withdraw from the study at any time and that their decision not to participate had no impact on their services. Participants were guaranteed confidentiality and they were assured as identifying information was not be made available to anyone. To ensure these, the information was identified using codes, and participants’ names were not used.

#### 2.2.1. The Life Satisfaction Index for the Third Age—Short Form (LSITA-SF12)

Life Satisfaction Index for the Third Age—Short Form (LSITA-SF12) was selected for cultural adaptation and investigation of psychometric properties. The tool is a 12-item scale on 6-point Likert response categories derived from the thirty-five-item LSITA, designed to measure the overall life satisfaction among elders, validated by Barrett, A.J [13]. For items 2 and 4–6, the responses are scored reversed as follows: Strongly disagree (6); Dis-agree (5); Slightly/partly disagree (4); Slightly/partly agree (4); Agree (2); and Strongly agree (1). For the rest of the items, 1, 3, 7–12, the responses are scored as follows: Strongly disagree (1); Disagree (2); Slightly/partly disagree (3); Slightly/partly agree (4); Agree (5); and Strongly agree (6). (Appendix A) Studies recommended Cronbach’s alpha value <0.60 as unacceptable, 0.60–0.64 undesirable, 0.65—0.69 minimally acceptable, 0.70–0.81 respectable, 0.82–0.90 very good, and much above 0.90 indicates redundancy that needs consideration of shortening the scale [14,15,16,17]. The 35-item instrument has a Cronbach alpha of 0.93. It was developed based on the theory of successful aging reported by Neugarten and Havighurst and their colleagues in the 1960s [18]. These aforementioned pioneer researchers developed the 20 item Life Satisfaction Index–Form A (LSI-A) [19] to measure successful aging. However, with the statistical improvement and measurement knowledge, an instrument with improved psychometric properties was needed; as a result, the LSITA-SF12 was developed. The reliability of the LSITA-SF12 scale was 0.90 with satisfactory content, construct, and criterion validity. It was developed and updated to measure the overall life satisfaction of elders [13].

#### 2.2.2. Translation and Cultural Adaptation of the Scales

In addition to the LSITA-SF12, the translation and cultural adaptation were performed for the sense of coherence (SOC), Katz Index of Independence in Activities of Daily Living (Katz index ADL), participation in activities (physical activities), Kessler Psychological Distress Scale (K10), satisfaction with life scale (SWLS), and Oxford happiness questionnaire (OHQ). The translations were performed according to the guideline for the process of cross-cultural adaptation of self-report measures [20].

Considering this guideline, the following steps were followed: An initial translation from English into the local language (Amharic) was performed by two independent English language experts and two Integrated Clinical and Community Mental Health experts who were native in the Amharic language and fluent in English. By consolidation of these language experts and medical experts, consensus was reached, and a preliminary initial translated version of the instruments in the target language, Amharic, was generated. The preliminary initial translated version was translated back into the original language by one English language expert and one Integrated Clinical and Community Mental Health and Clinical Psychology expert. Both experts were fluent English speakers with no knowledge about the original English versions of the scales. Then, the expert committee reviewed the translated version: The expert committee consisted of the investigators, Amharic and English language experts who performed the translation and back-translation, medical and surgical nursing specialists, sociologists, and public health experts. The expert committee compared back-translations with the original questionnaire and reviewed the grammatical structure of the sentences and similarity in meanings. Ambiguities and discrepancies regarding cultural meaning and phrases in words and sentences of the questionnaire were discussed and resolved through consensus of a pre-final version of the scales. Finally, to ensure comprehension, the pre-final Amharic versions of the scales were pre-tested. For the pre-test (psychometric debriefing), fifteen elders were asked to rate items using the scale (clear, somewhat clear, cannot decide, and unclear). To make the language clearer, they were also asked to rewrite the statements. After discussing with the experts’ committee, the final Amharic versions of the scales were created.

#### 2.2.3. Validation and Psychometric Properties of the Scales

Validity measurements of the Life Satisfaction Index for the Third Age—Short Form (LSITA-SF12):

Face validity: The face validity of the scale was assessed by ensuring the standard back-translation process, critical review, and expert panel opinions. Feasibility, readability, consistency of style and formatting, and the clarity of the language used were assessed by constructed questions.

Content validity: The content validity was measured by ensuring a standard back-translation process, literature review, expert panel opinions, and an expert content validity index (CVI). The CVI was assessed by experts and elderly people evaluations. Eight experts have assessed both the relevance of the items in terms of life satisfaction (irrelevant, cannot decide, somewhat relevant, and relevant) and the clarity (unclear, cannot decide, somewhat clear, and clear) of the items. Fifteen elderly people were asked to rate whether the items were unclear, undecided, somewhat clear, and clear.

Concurrent validity: The concurrent validity was computed by performing correlation analysis between theLSITA-SF12scale, and SWLS. SWLS was first developed by Diener et al. (1985) and adapted to different populations and countries as a measurement tool of life satisfaction [21]. The SWLS measures global life satisfaction and consists of 5 items where by the values are evaluated according to 7 scores (1 = strongly disagree, 7 = strongly agree). It has adequate criterion validity, good convergent, and discriminant validity and reliability has been demonstrated in terms of high internal consistency with a value of 0.87 and stability over time with a test-retest coefficient of 0.82.

Discriminant validity: The concept of happiness is related to life satisfaction. To check whether the LSITA-SF12 measures happiness, discriminant validity was computed between the LSITA-SF12 and the OHQ. The OHQ was developed by psychologists Michael Argyle and Peter Hills at Oxford University. It has 29 items with 6 categorical responses [22].To see the correlation of the dependent and selected independent variables/activity of daily living, mental health, and physical activity. Pearson correlation coefficients were calculated between LSITA-SF12 and Katz index ADL, K10, physical activity measurement scale, and sense of coherence scale, respectively.

##### Reliability Tests of the Life Satisfaction Index for the Third Age—Short Form (LSITA-SF12)

Internal consistency/internal validity: This is an important measurement property for questionnaires that intend to measure a single underlying concept (construct) by using multiple items. The internal validity of LSITA-SF12 was evaluated by Cronbach’s alpha coefficient.

Test–retest reliability: For the test-retest reliability, the second test/retest was done after two weeks of the first test. Inter-rater reliability: For inter-rater reliability, two data collectors were assigned to collect data separately among the same elderly participants.

### 2.3. Additional Measures

Before the psychometric test of the LSITA-SF12, validity tests were done for a sense of coherence, Katz index ADL, Participation in activities/physical activities, K10, SWLS, and OHQ. The finding showed excellence in feasibility, readability, consistency of styles, formatting, and language clarity of the scales. The internal consistency/Cronbach’s alpha of the scales was also found within the acceptable limit.

### 2.4. Quality Assurance Mechanisms

The questionnaire was piloted for psychometric debriefing one week before the actual data collection. To ensure the consistency of the collection technique and the acquisition of quality data, random checks were carried out by field supervisors and the principal investigator. Before the analysis, the collected data were checked for completeness and accuracy. In addition, after primary cleaning in Epi-data, all datasets were transferred to SPSS version 26 and double-checked.

### 2.5. Data Analysis

During and after data collection, the questionnaires were checked for completeness and consistency. The data were entered into Epi-data version 4.6.0.2 (The EpiData Association, Odense, Denmark) and then exported to and analyzed using SPSS version 26 (IBM SPSS, New York, NY, USA). Descriptive statistics were carried out to illustrate the cross-tabulation frequencies, percentages, means, and standard deviations. CVI was calculated in Excel for both experts and study participants. For experts, both individual items level (I-CVI) and scale level average CVI (S-CVI/Ave) were assessed for relevance and clarity of constructed items. For elderly people, I-CVI and scale level average CVI (S-CVI/Ave) were assessed only for clarity of constructed items. Concurrent validity, discriminant validity, and correlation analysis between outcome and selected explanatory variables were analyzed using Pearson correlation coefficients. Internal consistency (internal validity), test–retest reliability, and inter-rater reliability were computed using Cronbach’s alpha coefficient, Intraclass Correlation Coefficient (ICC), and Kappa statistics, respectively.

## 3. Results

### 3.1. Sociodemographic Characteristics of Study Participants

One hundred thirty (130) elderly people participated in this validation study. The mean age of respondents was 70.25 years (SD = ±8.71). More than half, 70 (53. 8%), of the respondents were female and 76 (58.5%) were born in urban areas. Sixty (45.2%) of the respondents were married and 52 (44.4.0%) of the participants had 1–3 children. Forty-five (34.6%) of the participants were unable to read and write and the majority (84.6%) were Orthodox Christians by religion. Fifty-four (41.5%) of the participants were living with children/grandchildren (Table 1).

### 3.2. Validity Findings

Face validity: The face validity of the LSITA-SF12 was found excellent in terms of feasibility, readability, consistency of styles, formatting, and clarity of the language.

Content validity: I-CVI among experts was found to range from 0.94–1.00 for relevance and 0.91–1.00 for clarity. The S-CVI/Ave of theLSITA-SF12 was 0.96 for relevance and 0.98 for clarity of items among experts. The I-CVI and S-CVI/Ave about the clarity of items among the participants were found to range from 0.91 to 1.00 and 0.94, respectively.

Concurrent validity: The concurrent validity was done between the LSITA-SF12 scale and the SWLS. The Pearson correlation coefficient of the two scales was 0.719, *p* < 0.001 (Table 2).

Discriminant validity: The discriminant validity was computed between the LSITA-SF12 and the OHQ. The Pearson correlation coefficient between these two scales revealed a score of 0.192, *p* = 0.028 (Table 3).

Correlation analysis: The findings showed that daily activity performance, sense of coherence, and participation in activities were positively correlated with life satisfaction. However, psychological distress was negatively correlated with life satisfaction (Table 4).

### 3.3. Reliability Findings

Internal consistency (internal validity): The Cronbach’s Alpha of LSITA-SF12 was 0.901. The mean and standard deviation of the scale were 45.08 and 12.387, respectively (Table 5).

Test-retest reliability: The average inter-class correlation coefficient of the test-retest of LSITA-SF12 revealed a score of 0.915, *p* < 0.001, 95% CI = 0.88–0.94 (Table 6).

Inter-rater reliability: For inter-rater reliability of LSITA-SF12, Kappa statistic was done and was found to be 0.788, *p* <0.001 (Table 7).

## 4. Discussion

This study reported on the cultural adaptation and psychometric properties of the Amharic version of LSITA-SF12 when used in elderly people in metropolitan cities of northwest Ethiopia. The report is compliant with the relevant reporting guidelines for this type of research (https://www.equator-network.org/?post_type=eq_guidelines&eq_guidelines_study_design=reliability-and-agreement-studies&eq_guidelines_clinical_specialty=0&eq_guidelines_report_section=0&s. accessed on 6 December 2021). Most importantly, the scale revealed satisfactory validity and reliability in terms of content, internal consistency, test-retest reliability, inter-rater reliability, and concurrent and discriminant validities.

The study had 130 elders, both male and female with a mean age and standard deviation of 70.25 years (SD = ±8.71). Participants with young–old to old–old, unable to read and write to first-degree educational status, and very good to very bad perceived health status were involved in the study. Even though participants were selected proportionally considering the above variations, 60% of them were between the ages of 60 to 69 years. This might be due to the lower proportion of the higher age groups from the general population. Participants of the study are considered to be representative of the elderly people living in the two metropolitan cities of northwestern Ethiopia in which the Amharic language, the official language of Ethiopia, is the dominant language in the area.

Concerning data quality, in psychometric debriefing, the items were found feasible, readable, consistent in style, formatting, and language clarity. To ensure the consistency of the data collection, supervisors monitored the fieldwork effectively. Before the analysis, the collected data were checked for completeness and accuracy. These data-quality techniques were crucial for cultural adaptation and also significant for the correctness of the psychometric tests.

The LSITA-SF12 has relevance and clear items and an excellent content validity index as confirmed in both individual and scale content validity index. The findings were excellent and consistent with other studies in which an item whose I-CVI value is >0.79 considered relevant; if it is between 0.70 and 0.79, the item needs revisions; if <0.7 the item should be eliminated. For S-CVI/Ave, if it is ≥0.9, the scale has an excellent content validity [23,24,25].

The Pearson correlation coefficient for concurrent validity demonstrated a strong positive relationship of the LSITA-SF12with the SWLS. Similar reports were reported in the original English Version LSITA-SF12 and the 35 items LSITA [13,26]. The finding was also supported by literature in which Pearson correlation coefficient between 0 and ±0.3 represents weak relationship; between ±0.3 and ±0.7 moderate relationship; between ±0.7 and ±1.0 strong relationship [27,28]. SWLS is a commonly used scale that it meant to assess life satisfaction of a general population [21]; thus, the strong relation of the scales indicated the significance of the LSITA-SF12 for the evaluation of life satisfaction.

The discriminant validity between the LSITA-SF12 and the OHQ was confirmed as the two scales measured different concepts. The finding is consistent with the literature in which the Pearson correlation coefficient between 0 and ±0.3 shows a week relationship of the scales [27,28]. Theoretically, life satisfaction has a similar concept to happiness but is not the same [29]. This finding demonstrated that the two scales are positively correlated but measure different concepts.

The Pearson correlation analysis on life satisfaction and the selected independent variables, such as daily activity performance, sense of coherence, and participation in activities showed a positive correlation between the two. Nevertheless, mental disorder as measured in the K10 Scale was negatively correlated with life satisfaction. K10 predicts the likelihood of mental disorders. Accordingly, a low value indicates mental wellness and a high value likely points out severe mental disorder. Thus, the negative association of the scales might be due to the interpretation of the scales [30]. The negative association of mental disorder and life satisfaction was also demonstrated in another study [31]. As literature showed, the PCC 0 indicates no linear relationship; +1/–1 = perfect positive/negative linear relationship [27,28].

The Cronbach’s Alpha of LSITA-SF12 revealed as the scale had very good internal consistency. In the internal validity test, the corrected item-total correlation of item #2/I am now going through the worst time of my life/was 0.394 which is slightly lower than the recommended 0.4. Even if we had deleted this item, the model did not improve. So, we included it in the model. The Cronbach’s alpha of the current study was almost in line with the original English version, 0.90 [13], but slightly lower than the 35 items English version, 0.93 [18]. The similarity might be because the comparison is made with its original version. However, the difference might be that the 35 items version is old and domain-specific.

The very high correlation between the first and second tests indicates that the scale has high reproducibility and reliability. The finding is supported with literature in which interclass correlation coefficient of 0.00−0.24 indicates little correlation, 0.25–0.49 indicates low correlation, 0.50–0.69 indicates moderate correlation, 0.70–0.89 shows high correlation, and 0.90–1.00 indicates very high correlation [27,32].

The finding showed the substantial agreement between the two raters. Kappa value of <0 indicates no agreement, 0.001–0.20 indicates non to slight, 0.21–0.40 indicates fair, 0.41–0.60 indicates moderate, 0.61–0. 80 indicates substantial, and 0.81–1 indicates an almost perfect agreement [33]. The current inter-rater reliability agreed with the recommendation of the literature.

The minimum and maximum points of the LSITA-SF12 are 12 and 72, respectively. Regarding the current study population, the minimum and maximum points were 22 and 66, respectively. The mean and standard deviation of the Amharic version of LSITA-SF12 were 45.1 and ±12.4, respectively. The Skewness and Kurtosis were −0.03 and −1.19, respectively, which is within the acceptable limit for a normal distribution. Considering the mean and the standard deviation of the scale, the level of life satisfaction was: dissatisfied 25 (19.2%), moderately satisfied 74 (56.9%), and satisfied 31 (23.9%). Taking mean and standard deviation as the classification of life satisfaction was also in line with the original English version of LSITA-SF12 [13]. However, the scale did not show a psychometric property difference in terms of gender.

### 4.1. Strength and Limitations

This is the first study to be performed on life satisfaction among the Ethiopian elderly using the generic measure LSITA-SF12. The study had a representative sample of elderly men and women, young–old to old–old, unable to read and write to well educated, very good to very bad perceived health status. The study was conducted using the most commonly used language in the study area and the country. The data were collected in the participants’ residential homes. This enabled us to get sufficient time, completed data, and a high response rate. In addition, it inspired test-retest and inter-rater reliability tests.

Even though this study demonstrated satisfactory psychometric properties and internal validities, it has some limitations. Firstly, the study was done among Amharic speaking Ethiopian elders. It might not be representative of all Ethiopians other than Amharic speakers. Secondly, the study was limited to households in metropolitan cities of northwestern Ethiopia, which may not be representative of elders living in streets, religious places, temporary settlements, and rural residents. As studies showed, place of residence, living arrangements, and condition of a living environment influence life satisfaction [34,35]. Hence, the current study may not be generalizable to all elderly populations. Thirdly, since the study was cross-sectional, it cannot be determining a causal relationship. Fourthly, the study had a self-reported methodology which might be prone to social desirability bias. To control this bias, participants were interviewed in quiet and separate areas. They were also well informed about the purpose of the study.

#### 4.1.1. Clinical Implications of the Study to the Nursing Profession

Globally, particularly in developed nations, nurses and nurse scientists have been leaders in elderly care and play critical roles in addressing the challenges. Nurse scientists conduct researches that inform evidence-based clinical interventions to promote health and manage illness in various health care settings and public facilities. As the nursing professionals constitute a majority off staff in many of the health institutions, nursing science will continue to build scientific evidence-based practice for improving clinical care and quality of life for the aging population [36]. By the same token, there is a direct relationship between life satisfaction and chronic diseases and other age-related health problems [37,38]. Therefore, standardizing the life satisfaction measurement scale and developing the Amharic version of the scale for the group of the elderly will be an input for nurses to manage the underlying health problems of elders, and it will contribute to the nursing evidence-based clinical practice at large to the nursing profession.

#### 4.1.2. Conclusion and Recommendation

The LSITA-SF12 was successfully translated into Amharic language, culturally adapted, and validated for Ethiopian elders. The finding demonstrated satisfactory evidence on the psychometric properties. The scale revealed an excellent face and content validity index and acceptable concurrent and divergent validities. It has also substantial internal consistency, test–retest reliability, and inter-rater reliability. The scale can be used for research and clinical purposes regarding Amharic speaking Ethiopian elders. Now, researchers, nurses, clinicians, community health care workers, and other concerned bodies can use the Amharic version LSITA-SF12 with great confidence.

## Figures and Tables

**Table 1:** Sociodemographic characteristics of elders living in metropolitan cities of northwest Ethiopia, 2020 (*n* = 130).

Variable	Frequency	Percent
Sex		
Male	60	46.2
Female	70	53. 8
Age (In the year)		
60–69	78	60.0
70–79	29	22.3
≥80	23	17.7
Residency		
Bahir Dar city	70	53. 8
Gondar city	60	46.2
Place of birth/grownup		
Urban	54	41.5
Rural	76	58.5
Marital status		
Married and currently have a partner	60	46.2
Widowed	51	39.2
Divorced	19	14.6
Do you have children?		
Yes	117	90
No	13	10
Number of children/*n* = 117/		
1–3	52	44.4
4–6	50	42.8
>6	15	12.8
Family size		
1–3	30	23.1
4–6	70	53. 8
>6	30	23.1
Educational status		
Unable to read and write	45	34.6
Able to read and write	37	28.4
Grade 1–8	21	16.2
Grade 9–12	14	10. 8
Certificate and above	13	10.0
Religion		
Orthodox Christian	110	84.6
Muslim	20	15.4
Do you have religious practice?		
Yes, always	89	68.5
Yes, sometimes	25	19.2
Yes, occasionally	16	12.3
Current occupation		
Retired	43	33.1
Non-governmental	3	2.3
Housewife	37	28.5
Merchant	12	9.2
Private	11	8.5
Farmer	2	1.5
Non-employed	22	16.9
Occupation before age 60 years		
Governmental	41	31.5
Housewife	38	29.2
Merchant	20	15.4
Private	21	16.2
Farmer	10	7.7
Current living condition		
Live alone	13	10.0
Live with partner	5	3. 8
Live with children/grandchildren	54	41.5
Live with partner and children/grandchildren	47	36.2
Live with relatives	2	1.6
Live with a partner, children/grandchildren, and relative	9	6.9
Perceived health status		
Very good	16	12.3
Good	42	32.3
Average	46	35.4
Bad	19	14.6
Very bad	7	5.4

**Table 2:** The concurrent validity between 12 items Life Satisfaction Index for the Third Age—Short Form (LSITA-SF12) and the satisfaction with life scale (SWLS).

Correlations			
		LSITA-SF12	SWLS
LSITA-SF12	Pearson Correlation	1	0.719 **
	Sig. (2-tailed)		0.000
	N	130	130
SWLS	Pearson Correlation	0.719 **	1
	Sig. (2-tailed)	0.000	
	N	130	130

** Correlation is significant at the 0.01 level (2-tailed).

**Table 3:** The discriminant validity between 12 items Life Satisfaction Index for the Third Age—Short Form (LSITA-SF12) and the Oxford Happiness Questionnaire (OHQ).

Correlations			
		LSITA-SF12	OHQ
LSITA-SF12	Pearson Correlation	1	0.192 *
	Sig. (2-tailed)		0.028
	N	130	130
Oxford Happiness Questionnaire	Pearson Correlation	0.192 *	1
	Sig. (2-tailed)	0.028	
	N	130	130

* Correlation is significant at the 0.05 level (2-tailed).

**Table 4:** The correlation between 12 items Life Satisfaction Index for the Third Age—Short Form (LSITA-SF12) and Katz index ADL, sense of coherence scale, participation in activities scale, and K10.

Correlations			
		LSITA-SF12	Katz index ADL
LSITA-SF12	Pearson Correlation	1	0.284 **
	Sig. (2-tailed)		0.001
	N	130	130
Katz index ADL	Pearson Correlation	0.284 **	1
	Sig. (2-tailed)	0.001	
	N	130	130
		LSITA-SF12	Sense of coherence scale
LSITA-SF12	Pearson Correlation	1	0.567 **
	Sig. (2-tailed)		0.000
	N	130	130
Sense of coherence scale	Pearson Correlation	0.567 **	1
	Sig. (2-tailed)	0.000	
	N	130	130
		LSITA-SF12	Participation in activity scale
LSITA-SF12	Pearson Correlation	1	0.539 **
	Sig. (2-tailed)		0.000
	N	130	130
Participation in activity scale	Pearson Correlation	0.539 **	1
	Sig. (2-tailed)	0.000	
	N	130	130
		LSITA-SF12	K10
LSITA-SF12	Pearson Correlation	1	−0.611 **
	Sig. (2-tailed)		0.000
	N	130	130
K10	Pearson Correlation	−0.611 **	1
	Sig. (2-tailed)	0.000	
	N	130	130

** Correlation is significant at the 0.01 level (2-tailed).

**Table 5:** Internal consistency (internal validity) of the LSITA-SF12.

Reliability Statistics				
Cronbach’s Alpha				N of Items
0.901				12
**Item-Total Statistics**				
Items	Scale Mean if Item Deleted	Scale Variance if Item Deleted	Corrected Item-Total Correlation	Cronbach’s Alpha if Item Deleted
1. Things are better and way different from what I expected them to be while I was a child.	41.54	123.847	0.723	0.888
2. I am now going through the worst time of my life.	40.82	136.896	0.394	0.905
3. I am happy as I was young/adult.	41.51	127.353	0.735	0.888
4. I would have been happier If my life had not been boring.	41.74	130.551	0.577	0.896
5. I could have been happier with my life than I am now.	41.60	133.405	0.498	0.900
6. The things I do now are uninteresting/boring.	41.05	133.679	0.513	0.899
7. I hope my next life would be better.	41.33	129.944	0.618	0.894
8. The things I do now are interesting as they were before.	41.69	126.571	0.750	0.887
9. I am happy with my life.	41.17	126.343	0.751	0.887
10. Everything is now interesting.	41.51	127.926	0.728	0.888
11. I am satisfied with my past life.	40.77	135.528	0.498	0.899
12. I am happy about everything I do.	41.12	128.977	0.713	0.889
**Scale Statistics**				
Mean	Variance	Std. Deviation	N of Items	
45.08	153.436	12.387	12	

**Table 6:** Test-retest reliability of the 12 items Life Satisfaction Index for the Third Age—Short Form (LSITA-SF12).

Intraclass Correlation Coefficient							
	Intraclass Correlation	95% Confidence Interval		F Test with True Value 0			
		Lower Bound	Upper Bound	Value	df1	df2	Sig
Single Measures	0.844 ^a^	0.786	0.887	11.885	129	129	0.000
Average Measures	0.915 ^b^	0.880	0.940	11.885	129	129	0.000

The two-way mixed-effects model where people effects are random and measures effects are fixed. ^a^ The estimator is the same, whether the interaction effect is present or not. ^b^ Type A intraclass correlation coefficients using an absolute agreement definition.

**Table 7:** Inter-rater reliability of the 12 items Life Satisfaction Index for the Third Age—Short Form (LSITA-SF12).

Symmetric Measures					
		Value	Asymptotic Standard Error ^a^	Approximate T ^b^	Approximate Significance
Measure of Agreement	Kappa	0.788	0.093	14.484	0.000
N of Valid Cases		20			

^a^ Not assuming the null hypothesis. ^b^ Using the asymptotic standard error assuming the null hypothesis.

## Data Availability

The summary data are available in the main document and the dataset analyzed will be available from the corresponding author on reasonable requests.

## Abbreviations

CI: Confidence interval, CVI: Content validity index, I-CVI: Item level content validity index, Katz index ADL: Katz Index of Independence in Activities of Daily Living, K10: Kessler Psychological Distress Scale, LSITA-SF12: The 12 items Life Satisfaction Index for the Third Age—Short Form, OHQ: Oxford happiness questionnaire, S-CVI/Ave: Scale level average content validity index, SOC: Sense of coherence, SD: Standard deviation, SWLS: Satisfaction with life scale, WHO: World Health Organization offices of the Bahir Dar and Gondar town for their permission letter and data collectors and supervisors for their commitment and the study participants for their valuable information.

## Appendix A

**Table A1:** The validated and psychometric tested the Amharic version of the Life Satisfaction Index for the Third Age—Short Form (LSITA-SF12).

Items (English vs. Amharic Language)	Alternative Answers					
	Strongly Disagree በጣምአልስማማም /1/	Disagree አልስማማም /2/	Slightly/Partly Disagree በተወሰነመልኩአልስማማም /3/	Slightly/Partly Agree በተወሰነመልኩእስማማለሁ /4/	Agree እስማማለሁ /5/	Strongly Agree በጣምእስማማለሁ /6/
1. Things are better and way different from what I expected them to be while I was a child. 1. እያደግሁ ስመጣ ነገሮች በልጅነቴ ካሰብኩት በላይ የተሻሉ ሆነዉ አገኘኋቸዉ፡፡						
2. I am now going through the worst time of my life. 2. ይህ በሕይወቴ በጣም የተከፋሁበት ጊዜ ነዉ፡፡						
3. I am happy as I was a young/adult 3. ልክ እንደ ወጣትነቴ/ጎልማሳነቴ አሁንም ደስተኛ ነኝ፡፡						
4. I would have been happier If my life had not been boring. 4. ኑሮየ አሰልቺ ባይሆን ኑሮ የበለጠ ደስተኛ ሕይወት ይኖረኝ ነበር፡፡						
5. I could have been happier with my life than I am now. 5. ከአሁኑ የተሻለ በሕይወቴ ደስተኛ ልሆን እችል ነበር፡፡						
6. The things I do now are uninteresting/boring. 6. የምሰራቸዉ ስራዎች የማያስደስቱ ወይም አሰልች ናቸዉ፡፡						
7. I hope my next life would be better. 7. የወደፊት ሕይወቴ ጥሩና አስደሳች ይሆናል ብየ ተስፋ አደርጋለሁ፡፡						
8. The things I do now are interesting as they were before. 8. አሁን የምሰራቸዉ ነገሮች ሁሉ ልክ እንደበፊቱ አስደሳች ናቸዉ፡፡						
9. I am happy with my life. 9. በሕይወቴ ደስተኛ ነኝ፡፡						
10. Everything is now interesting. 10. አሁን ላይ ሁሉም ነገር አስደሳች ነዉ፡፡						
11. I am satisfied with my past life. 11. ያሳለፍኩትን ሕይወትሳስብ እረካሁ፡፡						
12. I am happy about everything I do. 12. በምሰራቸዉ ስራዎች ሁሉ ደስተኛ ነኝ፡፡						

Note: For items 2, 4, 5, and 6, the responses are reversed scored as below; Strongly disagree (6), Disagree (5), Slightly/partly disagree (4), Slightly/partly agree (4), Agree (2), Strongly agree (1). For all other items, 1,3, 7–12, the responses are scored as below; Strongly disagree (1), Disagree (2), Slightly/partly disagree (3), Slightly/partly agree (4), Agree (5), Strongly agree (6).
